# Digital phenotyping and sociological perspectives in a Brave New World

**DOI:** 10.1111/add.13805

**Published:** 2017-05-04

**Authors:** Melanie Lovatt, John Holmes

**Affiliations:** ^1^ Faculty of Social SciencesUniversity of StirlingStirlingUK; ^2^ School of Health and Related ResearchUniversity of SheffieldSheffieldUK

**Keywords:** Alcohol, digital health technologies, drinking guidelines, ethics, lay epidemiology, public health, sociology

Skinner *et al.*'s Journal Club paper 1 was prompted by our original publication in *Addiction* 2. There are areas covered which deserve further discussion and in writing this, we find ourselves in two minds. While we agree with Skinner *et al.* that the development of drinking guidelines and other interventions could benefit from a more contextualized understanding of alcohol use, we feel that some of our key arguments and the problems that we sought to highlight may not have been appreciated fully. Moreover, Skinner's proposals raise new ethical challenges which merit attention. Here we discuss these points in turn.

## Measurement in Context

From a scientific perspective, any attempt to improve the measurement of behaviour is generally welcome. This is especially true for alcohol use, which is underestimated by as much as 60% in standard surveys (although measurement error is only part of the problem) and is too often treated as a homogeneous activity varying only in quantity and frequency 3. In reality, drinking covers a complex set of practices with both distinct and overlapping contexts, motivations, temporalities, rituals and embodied experiences 4. Thus, the potential for a single drinking guideline to be an active preventative ingredient across all these practices seems questionable, at best.

Skinner *et al.*'s paper promises innovative ways of harnessing smart phones, global positioning system (GPS) tracking and wearable technology, which would facilitate behaviour change techniques that capture bodily actions and account more accurately for the drinking location. Interventions sensitive to the drinking context may offer particular benefits, as a message sent to a student before an evening's heavy drinking with friends is unlikely to have the same relevance to a middle‐aged couple sharing a couple of bottles of wine and watching Netflix. However, if ‘precision prevention’ is to be realized, further technological refinement will be required. Simply recognizing the location in which drinking is taking place does not acknowledge the variation in drinking occasions, be they in the home or in the pub, and the differences between occasions are likely to matter to both participants and those around them 5.

## Locating the Social

Having acknowledged the potential benefits of a more precise and contextually aware behavioural science, we feel that much is still missing from this Brave New World, and at the centre of our concern is the absence of the social. In our paper we stressed that drinking is an inherently social practice and that individualized interventions which do not speak to social norms and understandings of drinking are likely to be limited in their effectiveness 2. While Skinner *et al.*'s proposals may include interventions utilizing a social norms approach, consideration is required as to how these norms may vary by context and, further, how this may be captured and harnessed to achieve public health gains. Understanding alcohol use *in situ* must go beyond collecting quantifiable data on the time, location and physical effects of drinking (e.g. blood alcohol concentration) to also understand what makes this kind of drinking meaningful and, therefore, ‘worth the risk’ 6, 7, 8, 9.

A further concern is the individualized approach to understanding risk. The technologies and intervention approaches described are rooted firmly in a traditional epidemiological approach which conceptualizes risk with reference to the individual. In contrast, the lay epidemiological theories we advanced argue that risk is constructed collectively with reference to experiences, observation of others' lives and both public and private discourse 10, 11. Giving someone more detailed and tailored data about their drinking might inform decision‐making, but will not necessarily result in behaviour change if the individual's perceptions of risk are constructed with reference to considerations beyond their own behaviour. As our paper showed, people's risk perceptions are not based simply on how much they have drunk and how this compares to recommended guidelines, but on how much they are drinking compared with people they know, examples of those who have suffered alcohol‐related harm and their personal experiences, both good and bad, of drinking ‘too much’. This echoes a wealth of previous qualitative studies, particularly on young adults' drinking, which highlight that information about the quantified amount of alcohol consumed is of less importance than the sensations experienced, the maintenance of a shared intoxication level by the drinking group and the behaviours of those who failed to ‘hold their drink’ or ‘keep up with the pace’ 8, 12, 13.

We also highlighted that lay epidemiological approaches consider health in the context of other issues, such as leisure, work and family life 14. For instance, the participants in our study acknowledged the possibility of negative health consequences from drinking, but balanced these against perceived benefits, such as socializing and ‘having a good time’. Other researchers have highlighted evidence that transgression of health guidance or other social norms is sometimes the point of drinking for both young and old 6, 15, 16. Additionally, when our respondents moderated their alcohol consumption this was not necessarily out of health concerns, but because of family and work responsibilities. Health professionals and researchers seeking to use digital technologies to change behaviour and improve health should take account of the benefits and pleasures of particular drinking contexts and how these connect to wider aspects of people's lives. Thus, more precise prevention does not mean accounting for just the spatial and temporal context but also the broader life context. More research is needed to understand how the kinds of personalized digital interventions discussed by Skinner *et al.* may be incorporated into everyday life and set in the context of people's responsibilities, pleasures and values. This may not only allow better understanding of their effects and limitations but may also permit tapping into factors which influence people's drinking behaviours, such as wanting to avoid being hungover in order to enjoy spending quality time with friends or family the next day.

## Technology, Ethics and Inequalities

The points made above concern the need to avoid overly individualized thinking when seeking to use digital interventions to improve individual drinkers' health. However, sociologists have also begun to ask broader questions about the ways in which these technologies shape our understandings of health and illness, and the social, ethical and moral implications of this.

First, an increased monitoring of everyday life‐styles by the individual, external observers or computer algorithms contributes to a paradigm shift in how health, and what it means to be ‘healthy’, is conceptualized. Instead of health constituting the absence of disease, it becomes an ongoing process which is measured and surveilled continually 17. Research is needed to explore how this relates to existing lay epidemiological framings of health, which prioritize function, feeling and embodied experiences of pain or disability rather than external measurements 18, 19. In the words of sociologist Deborah Lupton, will people become more inclined to ‘trust the ‘numbers’ over physical sensations’? 20

Secondly, an increased ability to monitor and track bodily practices raises questions of power; namely, who profits from the use of such technologies and what are the consequences of this? By using health applications (apps) on our smartphones and new wearable technologies, people may feel empowered and in control of their health, whereas previously they may have relied more upon professional guidance although, again, more research is needed to understand how these new technologies intersect with other sources of information and guidance. More problematically, increased empowerment may prompt feelings of increased responsibility to manage one's own health and sharpen the moral hazard implicit within neoliberal discourses of personality responsibility for health 21, 22. A distinction may be drawn between the ‘good, responsible, self‐monitoring and healthy citizens’ and, conversely, the ‘bad, irresponsible, unhealthy citizens who do not monitor their measurements and fail to modify their own behaviour’. This risks potential stigmatization of those who do not use available technologies through choice, economic constraint or practical necessity, and feelings of guilt and shame among those who do so but fail to adhere to the suggestions made by their devices 22. Issues of power are also relevant in terms of who owns and has access to the—very personal—data that are collected. While people may choose to share intimate health information voluntarily on social networks in order to receive peer support, there is increasing concern that the terms and conditions under which big data companies collect, store, utilize and sell users' data are presented in formats that are incomprehensible to the average person 23. In summary, the use of digital health devices contributes to the increased surveillance of people's bodies and makes public intimate health information which previously would have been known to only the patient and their doctor 24.

Thirdly, while targeting tailored health promotion interventions at individuals and subgroups may bring advantages and increase the probable relevance of health guidelines, Lupton argues that this should not come at the expense of population‐wide attempts to reduce social inequalities:

By focusing on the individual, sending regular messages to encourage that person to exercise or eat well, these technologies reduce health problems to the micro, individual level. Such approaches do little, therefore, to identify the broader social, cultural and political dimensions of ill‐health and the reasons why people may find it difficult to respond to such messages 22.

Indeed, such technologies, particularly more expensive variants, may in some cases exacerbate health inequalities, given that their use tends to be higher among those who are richer and better educated 25.

## Future Directions in a Brave New World

We have attempted here to present a balanced discussion of the implications of using digital innovations in behaviour change interventions. While there is potential for these technologies to advance scientific knowledge, their use contributes to long‐standing debates within public health on the importance of the social, the ethics of intervention and the root causes of inequality. Technologies are not ‘neutral’ 20 and, depending on how they are used, can reinforce or challenge prevailing paradigms and ideas. Using digital devices in the ways suggested by Skinner *et al.* predominantly supports a conceptualization of health that is rooted in individual responsibility and behaviour change. We are not expressing outright opposition to these developments, and there is room within our research and practice for a mixed economy of disciplinary perspectives. However, cross‐disciplinary learning is essential to science's contribution to improving public health. Therefore, we seek instead to remind readers of the arguments advanced in our original paper regarding the need for drinking guidelines and associated interventions to look beyond the risk relationships, individualized data and psychological models which inform much behavioural analysis.

Our paper was written before the new UK lower risk drinking guidelines were announced, and it is reassuring that the Guideline Development Group make reference to it in their report 26. None the less, the same report suggests that the group relied heavily on pre‐specified definitions of low‐risk behaviour and quantified epidemiological risk relationships in selecting their guideline rather than a detailed consideration of how that guideline might function in the context of people's actual drinking practices (and we acknowledge that the authors of this response were involved to differing degrees in that process). Perhaps such an orientation is inevitable when faced with an overwhelmingly complex drinking culture, but it should also remind us that digital phenotyping and other improvements in quantification are not a substitute for fully incorporating sociological thinking within policy decisions around alcohol, public health and addiction.

### Declaration of interests

None.
